# Supplementary material for: The interactome of intact mitochondria by cross-linking mass spectrometry provides evidence for coexisting respiratory supercomplexes
Source: Mol Cell Proteomics. 2017 Dec 8;17(2):216–32. doi: 10.1074/mcp.RA117.000470 (PMC5795388; doi:10.1074/mcp.RA117.000470)
Supplement: Supplemental Data [file supp_RA117.000470_133922_0_supp_23978_fzffwf.zip › spectra_annotation/mito_spectra_annotation/177-1-9-1-21-1.pdf]

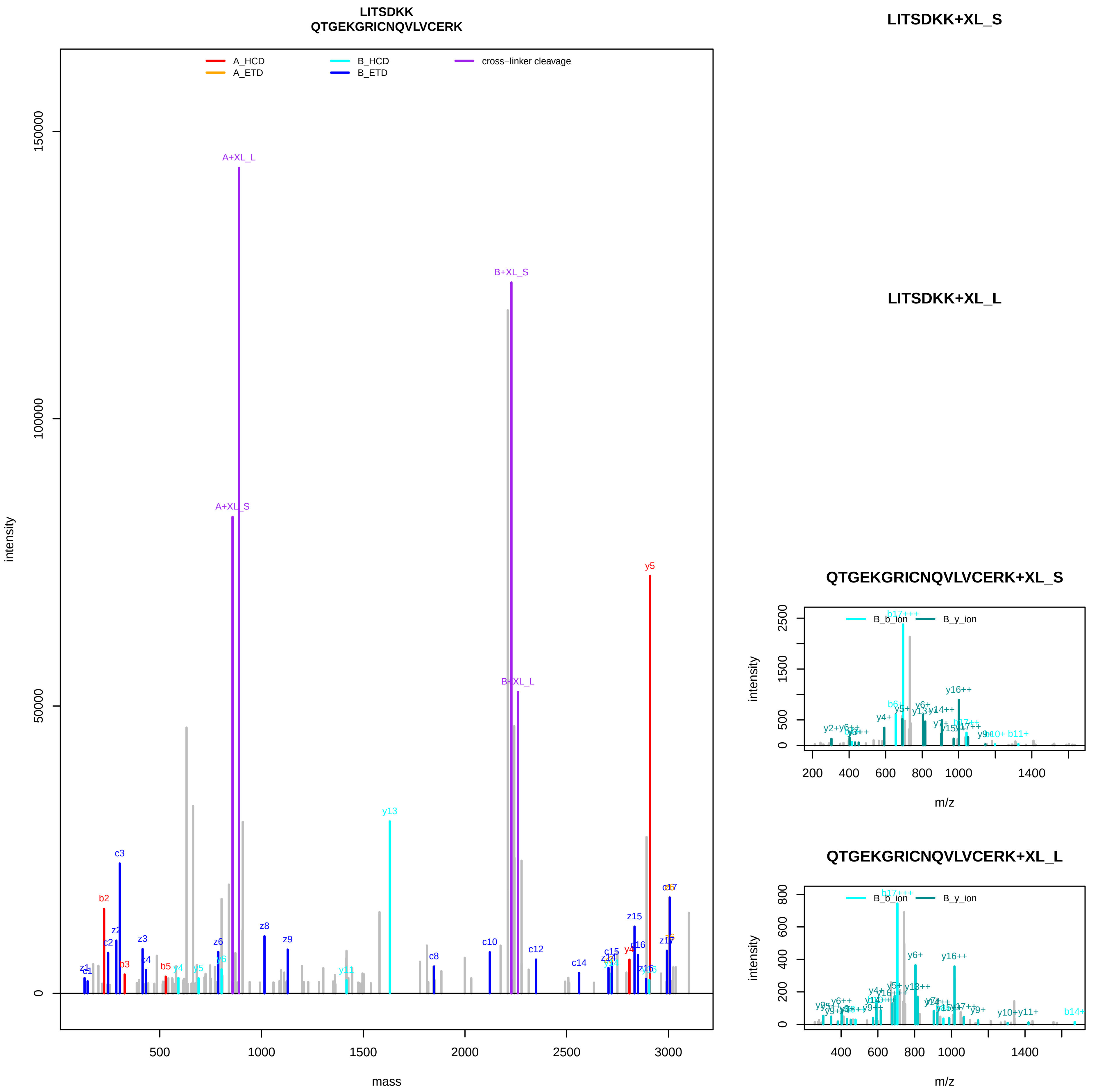
LITSDKK
QTGEKGRICNQVLVCERK
A_HCD
A_ETD
B_HCD
B_ETD
cross−linker cleavage
A+XL_L
A+XL_S
B+XL_S
B+XL_L
y5
y13
c3
b2
c2
z2
z3
c4
b3
b5
z6
z8
z9
c8
c10
c12
c14
z14
c15
z15
c16
z16
z17
c17
y4
y11
intensity
mass
0
50000
100000
150000
500
1000
1500
2000
2500
3000
LITSDKK+XL_S
LITSDKK+XL_L
QTGEKGRICNQVLVCERK+XL_S
B_b_ion
B_y_ion
b17+++
y16++
m/z
QTGEKGRICNQVLVCERK+XL_L
y6+
y16++
y13++
0
500
1500
2500
200
400
600
800
1000
1400
